# High-Fat-Diet-Induced Obesity Produces Spontaneous Ventricular Arrhythmias and Increases the Activity of Ryanodine Receptors in Mice

**DOI:** 10.3390/ijms19020533

**Published:** 2018-02-10

**Authors:** Gina Sánchez, Felipe Araneda, Juan Pedro Peña, José Pablo Finkelstein, Jaime A. Riquelme, Luis Montecinos, Genaro Barrientos, Paola Llanos, Zully Pedrozo, Matilde Said, Ricardo Bull, Paulina Donoso

**Affiliations:** 1 Programa de Fisiopatología, Instituto de Ciencias Biomédicas, Facultad de Medicina, Universidad de Chile, 8380453 Santiago, Chile; ginaluisa.sanchez@gmail.com; 2 Programa de Fisiología y Biofísica, Instituto de Ciencias Biomédicas, Facultad de Medicina, Universidad de Chile, 8380453 Santiago, Chile; felipe343@hotmail.com (F.A.); jfinkeht@med.uchile.cl (J.P.F.); montecinosl@yahoo.com (L.M.); gcbarrientos@gmail.com (G.B.); zpedrozo@med.uchle.cl (Z.P.); rbull@med.uchile.cl (R.B.); 3 Escuela de Ciencias Veterinarias, Universidad de Viña del Mar, 2572007 Viña del Mar, Valparaíso, Chile; jppenam@outlook.com; 4 Advanced Center for Chronic Diseases, Facultad de Ciencias Químicas y Farmacéuticas, Universidad de Chile, 8380494 Santiago, Chile; jriquelme43@gmail.com; 5 Instituto de Investigación en Ciencias Odontológicas, Facultad de Odontología, Universidad de Chile, 8380492 Santiago, Chile; pllanos@odontologia.uchile.cl; 6 Centro de Investigaciones Cardiovasculares, CCT-CONICET La Plata, Facultad de Medicina, Universidad Nacional de La Plata, 1900 La Plata, Argentina; msaid@med.unlp.edu.ar

**Keywords:** calcium release channels, reactive oxygen species (ROS), redox modifications, ventricular tachycardia, NADPH oxidase

## Abstract

Ventricular arrhythmias are a common cause of sudden cardiac death, and their occurrence is higher in obese subjects. Abnormal gating of ryanodine receptors (RyR2), the calcium release channels of the sarcoplasmic reticulum, can produce ventricular arrhythmias. Since obesity promotes oxidative stress and RyR2 are redox-sensitive channels, we investigated whether the RyR2 activity was altered in obese mice. Mice fed a high fat diet (HFD) became obese after eight weeks and exhibited a significant increase in the occurrence of ventricular arrhythmias. Single RyR2 channels isolated from the hearts of obese mice were more active in planar bilayers than those isolated from the hearts of the control mice. At the molecular level, RyR2 channels from HFD-fed mice had substantially fewer free thiol residues, suggesting that redox modifications were responsible for the higher activity. Apocynin, provided in the drinking water, completely prevented the appearance of ventricular arrhythmias in HFD-fed mice, and normalized the activity and content of the free thiol residues of the protein. HFD increased the expression of NOX4, an isoform of NADPH oxidase, in the heart. Our results suggest that HFD increases the activity of RyR2 channels via a redox-dependent mechanism, favoring the appearance of ventricular arrhythmias.

## 1. Introduction

Sudden cardiac death (SCD) represents a high proportion of all natural deaths [1]. Ventricular arrhythmias, such as ventricular tachycardia and ventricular fibrillation, that interfere with the normal blood pumping activity of the heart, are prevalent causes of SCD, and obese subjects are at a higher risk of SCD than non-obese subjects [2,3,4]. Obesity is frequently associated with dyslipidemia, insulin resistance, and type 2 diabetes, and all these conditions further increase the risk of arrhythmias [5]. Cardiac contraction is initiated by an action potential that spreads through the membrane of the cardiomyocytes. The duration and shape of the ventricular action potential depends on the coordinated interplay of multiple outward and inward ionic currents. Changes in electrochemical gradients, or abnormal gating of any of the ion channels responsible for these currents, can be involved in the generation of arrhythmias.

Disruption of calcium homeostasis is involved in the pathogenesis of ventricular arrhythmias. Ryanodine receptors (RyR2), the sarcoplasmic reticulum (SR) calcium release channels, provide most of the calcium needed for heart contraction during systole, and their opening is tightly controlled by the L-type calcium current that enters the cell during the action potential. During diastole, RyR2 must close to allow for the reuptake of calcium into the SR [6]. Aberrant release of calcium during diastole, due to the failure of RyR2 to close properly, activates its extrusion via the sodium-calcium exchanger, generating a net inward current that can depolarize the cardiomyocyte cell membrane (delayed after depolarization) and cause arrhythmias. Genetic studies have shown that catecholaminergic polymorphic ventricular tachycardia (CPVT), a severe form of arrhythmia induced by emotional stress or exercise in patients without previous cardiac disease, is caused by missense mutations of the RyR2 gene that disrupt the interactions between the protein domains responsible for channel closing [7,8]. Post-translational modifications of RyR2 can also prevent the correct channel closing. RyR2 contains hyperreactive cysteines that are oxidatively modified by reactive oxygen species (ROS), especially in the presence of an imbalance of ROS in the cell [9]. In vitro oxidation of RyR2 increases the channel response to cytoplasmic calcium concentration and favors the calcium release in isolated cardiomyocytes, generating calcium waves and arrhythmias [10,11].

Obesity is characterized by an increased generation of ROS [12,13], which may impact RyR2 function and affect the heart rhythm. The RyR2 activity in the hearts of obese animals has not yet been investigated.

Feeding C57BL/6 mice with a high fat diet (HFD) for eight weeks is a well-established method to induce obesity [14]. Therefore, using this model, we studied the response to cytoplasmic calcium of single RyR2 channels incorporated in planar bilayers and quantified the occurrence of spontaneous arrhythmias through electrocardiogram (ECG) recordings. We also investigated the effect of apocynin on the activity of these channels. Apocynin (acetovanillone) is a cathecol-derived molecule produced in the roots of *Picrorhiza kurroa*, a plant used in traditional Indian medicine as an anti-inflammatory and antioxidant. Apocynin has ROS scavenging properties and is a potent inhibitor of NADPH oxidase, isoform 2 (NOX2), whose activity increases in several tissues in obesity and hyperglycemia [15,16,17]. We previously showed that apocynin prevents the increase in RyR2 activity caused by reversible redox modifications of the protein after a short episode of ischemia-reperfusion in rat hearts [18].

## 2. Results

### 2.1. Mice Body Weight, Glucose, and Plasma Lipids

Mice fed with an HFD became obese after eight weeks and had an average 35% increase in body weight with concomitant increases in fasting glucose and total cholesterol. Plasma triglycerides, however, were similar in both control and HFD-fed mice. The addition of apocynin to drinking water (1.5 mmol/L) during the last four weeks of treatment did not produce significant changes in these metabolic parameters (Table S1).

### 2.2. ECG Recording

**Figure 1:**
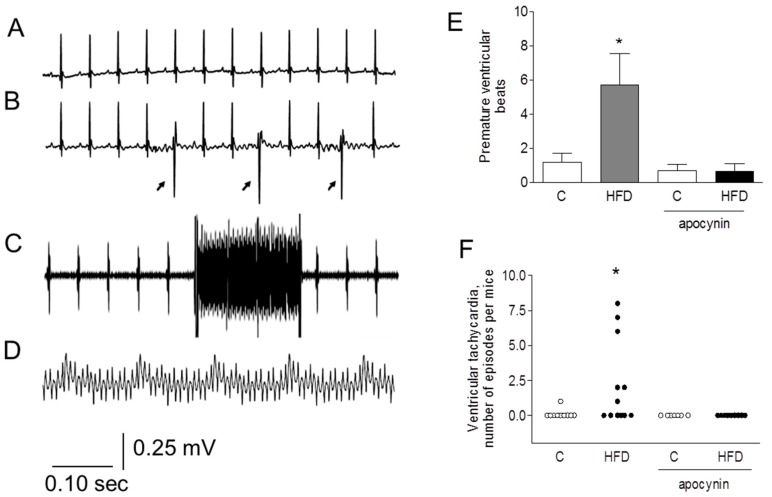
A high fat diet (HFD) increases the occurrence of spontaneous ventricular arrhythmias. Representative electrocardiographic records obtained in (**A**) control and (**B**–**D**) HFD-fed mice: (**A**) normal sinus rhythm, (**B**) premature ventricular beats (PVB, arrows), (**C**) non-sustained monomorphic ventricular tachycardia (VT), and (**D**) sustained bidirectional VT. (**E**) Average number of PVB during 5 min of recording in control (*n* = 11) and HFD-fed mice (*n* = 12), with or without apocynin. (**F**) Number of ventricular tachycardia (VT) episodes per mouse in control and HFD-fed mice, with or without apocynin. * *p* < 0.05 compared to all other conditions, Kruskal–Wallis test followed by Dunns test.

We recorded surface ECG (Lead I) under light anesthesia with isoflurane. A representative ECG record obtained in a mouse fed with the control diet is shown in Figure 1A. Control mice had normal sinus rhythms exhibiting only occasional or no ventricular extrasystoles during the six-minute recording period. Exceptionally, one mouse had a single episode of spontaneous ventricular tachycardia (VT), regaining normal rhythm after 1 s. In contrast, 6 out of 12 mice fed the HFD showed different arrhythmic events during the six-minute recording period. These events ranged from frequent ventricular extrasystoles (Figure 1B) to one or more episodes of monomorphic ventricular tachycardia (Figure 1C). Two mice had sustained ventricular tachycardia during most of the recording period: one with monomorphic ventricular tachycardia and another with bidirectional ventricular tachycardia (Figure 1D). Apocynin greatly reduced the number of premature ventricular beats, both in control and HFD-fed mice (Figure 1E) and completely eliminated the tachycardia events in HFD-fed mice (Figure 1F).

### 2.3. Echocardiographic Measurements

To assess the effect of eight weeks of HFD on cardiac function, we determined the fractional shortening of the left ventricle in echocardiograms obtained in conscious mice. Compared to controls, HFD-fed mice had a significantly higher heart rate, which was not changed by apocynin (Table 1), but the ventricular internal dimensions in systole or diastole were not different from controls, so no change was found in the shortening fraction, indicating a normal systolic function after eight weeks of HFD (Table 1).

### 2.4. Single RyR2 Channel Activity from Mouse Ventricular Muscle

**Figure 2:**
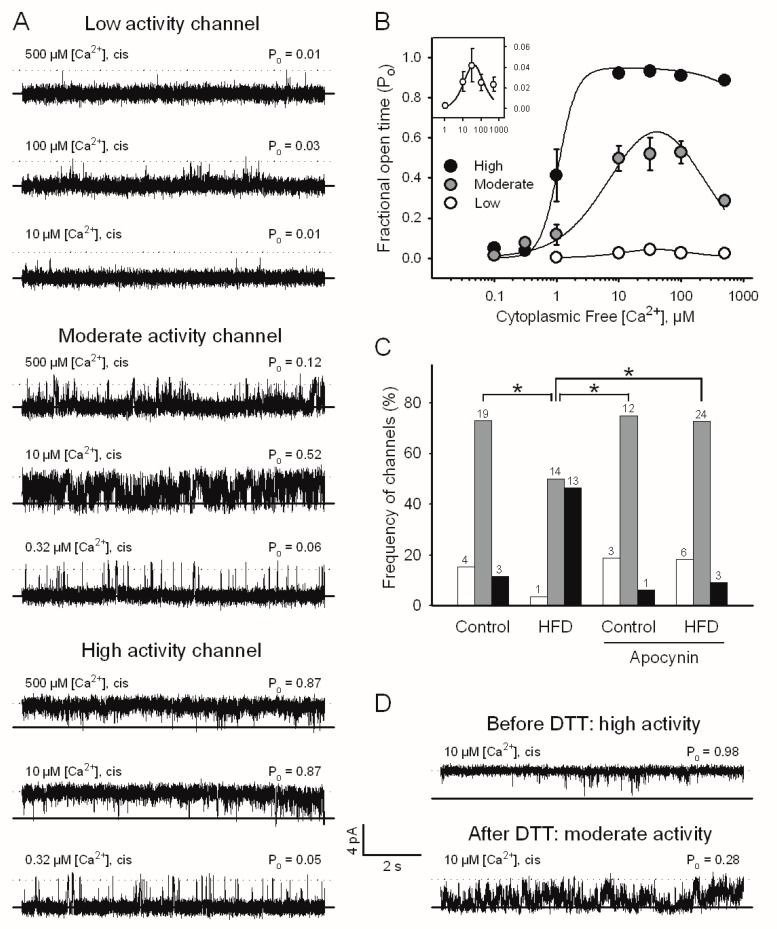
HFD increases the activity of single cardiac RyR2 channels in planar bilayers. (**A**) Representative current recordings show low, moderate, and high activity responses to Ca^2+^. Free Ca^2+^ concentrations ([Ca^2+^]) in the cytoplasmic compartment, and average P_o_ values, calculated from at least 30 s of continuous recordings, are given at the top left or right of each trace, respectively. The lipid bilayer was held at 0 mV. Channels open upward. The current is carried by Ca^2+^; (**B**) The Ca^2+^ response of low (open circles), moderate (gray circles), and high activity channels (black circles). Symbols and error bars depict mean ± SEM. The inset shows the low activity response with an amplified vertical scale. Solid lines represent the best non-linear fits to Equation 1 (see Section 4.6). Fitting parameters are depicted in Table 2; (**C**) The frequency of the incorporation of channels with low (open bars), moderate (gray bars), or high activity (black bars) from the hearts of control and HFD-fed mice drinking water without or with apocynin during Weeks 5–8. * *p* < 0.05, chi square test; (**D**) Representative current recordings of an RyR2 channel obtained from the heart of mice fed with an HFD without apocynin that spontaneously displayed high activity before (upper record) and after dithiothreitol (DTT) (lower record).

We incorporated single RyR2 channels from cardiac muscle of mice fed with HFD into planar bilayers to study their response to different cytoplasmic free Ca^2+^ concentrations ([Ca^2+^]). We previously showed that single RyR2 channels isolated from the hearts and brains of rats and rabbits respond to Ca^2+^ with low, moderate, or high activation, depending on the redox state of the protein [18,19,20]. RyR2 channels isolated from mice hearts exhibited the same responses to cytoplasmic [Ca^2+^] (Figure 2A,B). Low and moderate activity channels displayed a bell-shaped response to cytoplasmic [Ca^2+^] in the concentration range of 5 to 500 µM, reaching maximal P_o_ values of 0.05 and 0.5, respectively, at 30 µM [Ca^2+^] and significant inhibition at higher [Ca^2+^] (Figure 2B and Table 2). In contrast, high activity channels were activated near 1 µM [Ca^2+^], reached a P_o_ near 1.0 in the range of 5 to 500 µM [Ca^2+^], and showed no Ca^2+^-dependent inhibition (Figure 2B and Table 2). RyR2 channels obtained from control mice exhibited moderate activity with the highest frequency (Figure 2C). Out of the 26 channels from the control mice, 4, 19, and only 3 displayed low, moderate, and high activity. Of the 28 channels analyzed in mice fed an HFD, only one channel had low activity, 14 channels had moderate activity, and 13 channels showed high activity. Therefore, the HFD caused a significant shift in the distribution of RyR2 channel responses to cytoplasmic [Ca^2+^], favoring responses with higher channel activity (Figure 2C). The supplementation of drinking water with apocynin prevented the increased activity of RyR2 in HFD-fed mice, as only 3 out of 33 channels analyzed showed high activity, 24 had moderate activity, and 6 had low activity (Figure 2C). Apocynin did not change the distribution of channel responses to cytoplasmic [Ca^2+^] in mice fed the control diet (Figure 2C), suggesting a balanced redox state.

When high activity channels were incubated with dithiothreitol (DTT), a thiol reducing agent, when still incorporated in the bilayer, their activity at 10 µM [Ca^2+^] significantly decreased from a P_o_ value of 0.96 ± 0.02 to a P_o_ value of 0.46 ± 0.02, which corresponds to the moderate response of RyR2 channels (*p* < 0.05, *n* = 3). An example of such a channel is shown in Figure 2D. This result suggests that redox modifications of thiol residues are responsible for the increased frequency of the high activity response to cytoplasmic [Ca^2+^].

### 2.5. RyR2 Content and Phosphorylation

**Figure 3:**
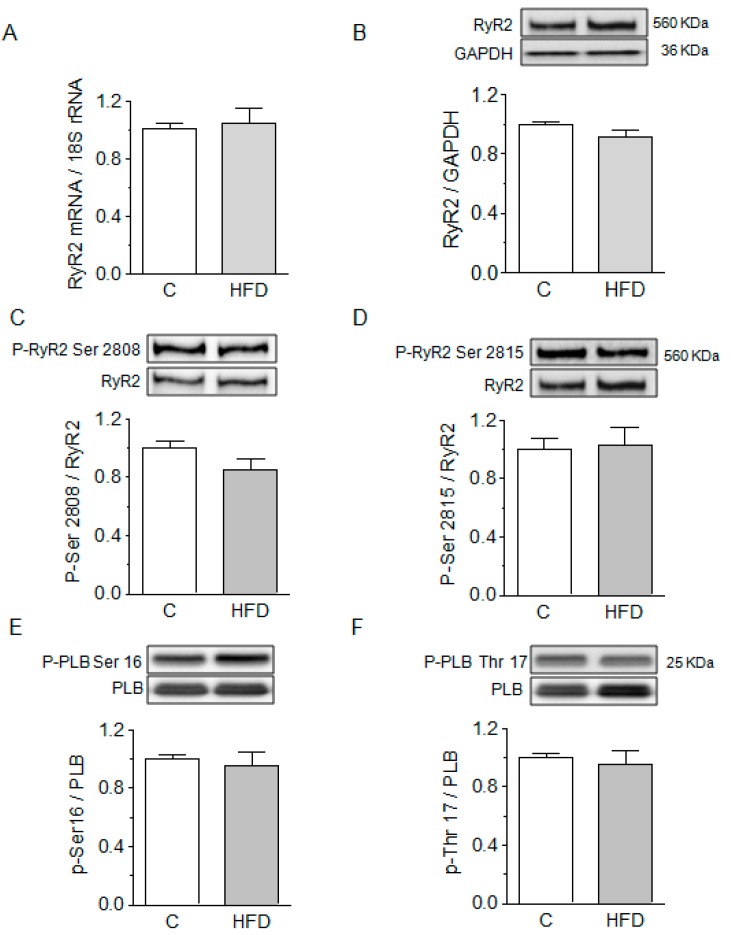
HFD does not modify RyR2 content or phosphorylation. (**A**) RyR2 mRNA determined by quantitative real-time polymerase chain reaction (qRT-PCR) (*n* = 6); (**B**–**D**) Representative Western blots of total and phosphorylated forms of RyR2 at ser-2808 or ser-2814; (**E**,**F**) Representative Western blots of total and phosphorylated phospholamban (PLB) at thr-17 or at ser-16. Bars represent the mean ± SEM of six determinations like those shown on top, obtained in different hearts.

Increases in RyR2 content or phosphorylation status could account for the ventricular arrhythmias observed in HFD-fed mice and the increased activity observed in isolated channels. However, we did not find changes in RyR2 mRNA expression or protein abundance in mice hearts after eight weeks of HFD (Figure 3A,B, respectively). Additionally, no changes occurred in the phosphorylation of RyR2 at Ser-2808 or Ser-2814 (Figure 3C,D), the PKA- and CaMKII-dependent sites, respectively [21,22,23]. These results suggest that neither increases in RyR2 content nor changes in its phosphorylation status play a role in the observed activation of RyR2. To confirm that no activation of PKA or CaMKII occurred in our mice, we determined the phosphorylation of phospholamban at Ser-16, the PKA site (Figure 3E) and Thr-17, the CaMKII site (Figure 3F). The unchanged phosphorylation state at these two sites suggests that no activation of these kinases occurred after eight weeks of HFD.

### 2.6. Effect of HFD on the Free Thiol Content of RyR2

**Figure 4:**
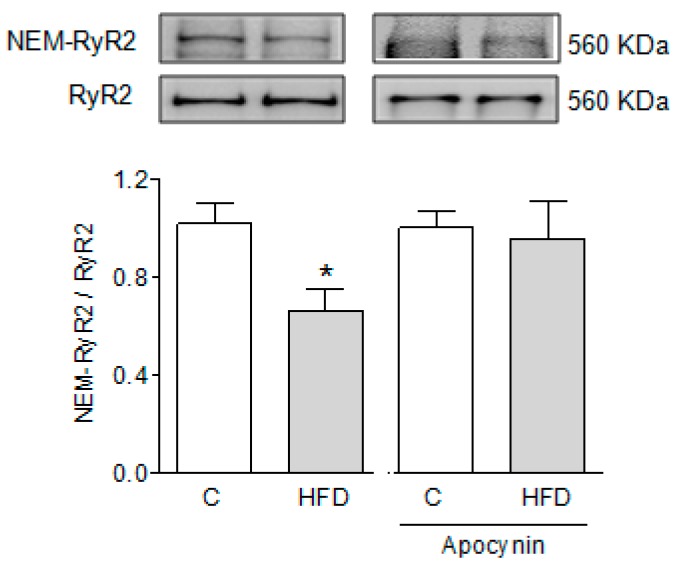
HFD decreases the content of free thiol residues in RyR2. Representative Western blots of NEM-biotin incorporation in RyR2 in control and HFD-fed mice. Bars represent the mean ± SEM of six determinations like those shown on top, obtained in six different sarcoplasmic reticulum (SR) fractions. Each SR fraction was prepared from a pool of three to five hearts. * *p* < 0.05 compared to controls, one-way analysis of variance (ANOVA), followed by Tukey test.

Because single channel experiments suggest a more oxidized RyR2 state, we determined the content of free SH residues of the protein by incubation with biotin labeled *N*-ethyl-maleimide (NEM-biotin). As shown in Figure 4, RyR2 channels from HFD-fed mice incorporated significantly less NEM-biotin than RyR2 channels from controls, indicating a significantly reduced number of free SH residues. Supplementation of drinking water with apocynin prevented the decrease in free thiol residues of RyR2 in HFD-fed mice (Figure 4).

### 2.7. NOX Isoforms in the Hearts of HFD-Fed Mice

**Figure 5:**
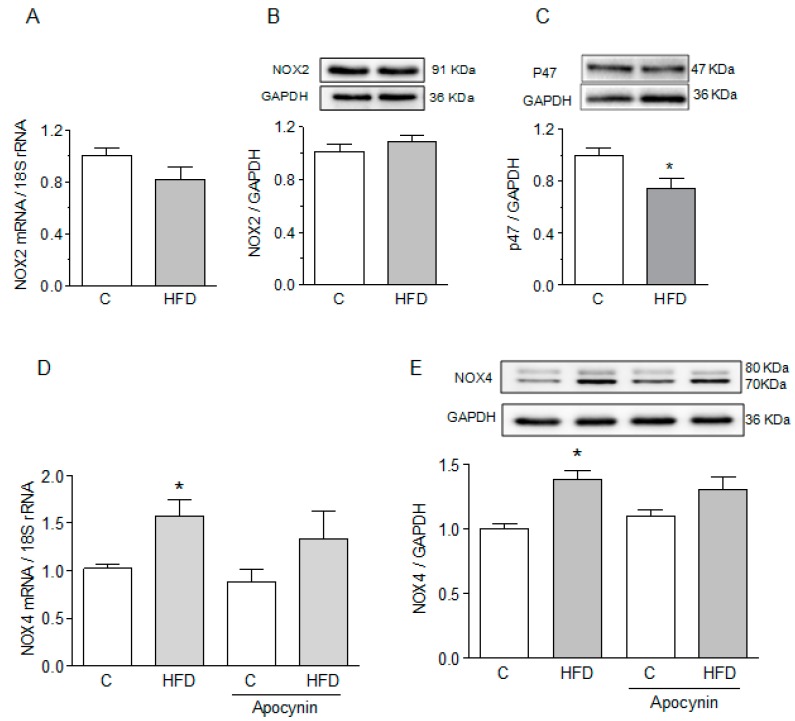
HFD increases the expression of NOX4 but not NOX2. (**A**) NOX2 mRNA determined by qRT-PCR. (**B**) Representative Western blots and quantification of NOX2 and (**C**) p47phox. (**D**) NOX4 mRNA determined by qRT-PCR in control and HFD-fed mice treated with or without apocynin, and (**E**) representative Western blots and quantification of NOX4, in control and HFD-fed mice treated with or without apocynin. Bars represent the mean ± SEM of six determinations obtained in different hearts. * *p* < 0.05 compared to controls, one-way ANOVA, followed by Tukey test.

To investigate whether the expression of NOX isoforms was altered in mice fed an HFD, we measured the mRNA expression of NOX2 by qPCR and the protein abundance in Western blots. We found no difference between HFD-fed mice and controls in the mRNA expression (Figure 5A) or content of this protein (Figure 5B). Likewise, the amount of p47phox, a regulatory cytosolic subunit of NOX2, associated with the membrane fraction decreased, suggesting that this subunit was not recruited to the membrane and therefore NOX2 was not activated in the hearts of HFD-fed mice (Figure 5C). In contrast, we found a significant increase in both mRNA (Figure 5D) and the protein content of NOX4 (Figure 5E) in HFD-fed mice, although neither mRNA nor protein content of NOX4 were substantially modified by apocynin.

## 3. Discussion

Obesity increases the generation of reactive oxygen species (ROS), which disrupts the cellular redox balance and leads to cardiac dysfunction including the generation of potentially life-threatening ventricular arrhythmias [4,12]. In this work, we observed a more frequent occurrence of arrhythmic episodes in HFD-fed mice associated with an enhanced response of single RyR2 channels to cytoplasmic [Ca^2+^]. Both the arrhythmic events and the higher activity of RyR2 were prevented by the addition of apocynin to the drinking water. This is the first direct demonstration of an HFD-dependent increase in RyR2 single-channel activity and its prevention with apocynin.

In this work, we also confirmed that RyR2 channels isolated from mice hearts displayed the same three types of response to Ca^2+^ observed in rat and rabbit hearts and brains. The HFD did not produce a new response to Ca^2+^ of RyR2 channels; instead, it favored the appearance of channels that responded to calcium with the highest activity, similar to that previously described in cerebral or heart global ischemia [18,20]. Since no changes occurred in the phosphorylation status of RyR2 that could account for its increased activity, the simplest way to explain this enhanced response is the redox-dependent modifications in SH residues of the RyR2 protein, which leads to a decrease in the [Ca^2+^] for half-maximal activation and an increased [Ca^2+^] for half-maximal inhibition, as has been previously reported [18,19,20]. The in vitro effect of DTT on channel activity and the decreased free thiol content of RyR2 support this idea.

A large amount of evidence, obtained in humans and animals, shows that oxidized RyR2 produces arrhythmias [24]. Obesity is characterized by increased oxidative stress due to the generation of ROS in various metabolic pathways [25,26]. NADPH oxidases are important sources of ROS in the heart. The heart expresses two isoforms of NADPH oxidase, NOX2 and NOX4. Whereas NOX2 requires the recruitment of several cytosolic subunits for activation [27], NOX4 is constitutively active and regulated at the transcriptional level of the protein [27]. To the best of our knowledge, the effects of an HFD in the activation of NOX in the heart have not been reported, but several studies show that NOX2 is activated in response to an HFD in endothelial cells [28], skeletal muscles [29], the brain [30], the kidney [31] and aortic smooth muscles [32]. Therefore, this isoform is considered to be responsible for the oxidative stress and damage induced by an HFD in these different tissues. In contrast to these reports, we found that NOX2 activity did not increase in the cardiac tissue of HFD-fed mice. No differences were found in the mRNA content or protein abundance of the NOX2 catalytic subunit, gp91phox, or in the association of the regulatory subunit p47phox to the membrane fraction, which would have been an indicator of activation. Instead, we found an increase in the mRNA and protein content of NOX4, which is the NADPH isoform that resides mostly in the internal membranes of the cell. The heart particularly responds to metabolic stress with a different isoform than most tissues, which could be inherent to heart function. The heart undergoes cyclic changes in volume and pressure, so its membranes are under continuously varying mechanical stress. We have previously shown that NOX2 is activated by rapid pacing or exercise in dog hearts [33,34], two conditions that infer a significant mechanical stress for cardiomyocytes. Additionally, NOX2 is rapidly activated within 1 min of reperfusion of rat hearts subjected ex vivo to 5 min of ischemia, but activation disappears after 5–15 min of reperfusion [18], suggesting that fast activation is followed by fast deactivation of the enzyme. In fact, the activation–deactivation of NOX2 may also occur on a beat-to-beat basis in isolated cardiomyocytes [35,36]. In the presence of a chronic stimulus, as in this work, in which the animals received an HFD for several weeks, different pathways might have been activated, resulting in the expression of a different isoform. Apocynin (4-hydroxy-3-methoxyacetophenone) is a naturally occurring anti-inflammatory compound that has been widely used as an NOX2 inhibitor in vivo because it inhibits the association of the regulatory cytosolic subunit p47phox to the membrane [15]. However, apocynin is also an antioxidant [16] and a peroxide scavenger [17]; therefore, via its ROS scavenger activity, apocynin can override the effects of both isoforms, NOX2 and NOX4.

Increased RyR2 activity, evidenced by increased sparks frequency, has been reported in the fructose-induced obesity model [37]. In this model, the authors demonstrated that the increased activity of RyR2 is due to CaMKII-dependent phosphorylation of the channel protein. In this work, we did not observe differences in the phosphorylation of RyR2 in serine 2814 in mice fed with an HFD compared to controls. Furthermore, we observed no increase in the phosphorylation of phospholamban at threonine 17, a classical target of CaMKII, suggesting that this kinase was not activated and therefore was not responsible for the increased RyR2 activity. Additionally, protein kinase A was not activated, evidenced by the lack of phosphorylation at serine 2808 of RyR2 and of phospholamban at serine 16. Rather, the activation of RyR2 is due to the oxidative modification of the protein in the hearts of HFD-fed mice, as suggested by the in vitro inhibitory effect of DTT on channel activity and by the significant decrease in the free thiol residues of RyR2.

Hyperreactive cysteines in RyR2 can undergo diverse redox modifications by reactive oxygen and nitrogen species [38]. In this study, we did not investigate the nature of the redox modification responsible for the increased RyR2 activity. We previously demonstrated that *S*-gluthationylation can reversibly activate RyR2-mediated Ca^2+^ fluxes in SR vesicles [33,34], and calcium channels in planar bilayers [18]. Likewise, *S*-nitrosylation also increases RyR2 activity [39]. Both RyR2 modifications, *S*-glutathionylation and *S*-nitrosylation, increase during reperfusion of ischemic hearts, and selective inhibition of any of them further increases the frequency of reperfusion arrhythmias [40], suggesting a protective effect against arrhythmias in the acute setting of ischemia-reperfusion. Therefore, it is unlikely that *S*-glutathionylation or *S*-nitrosylation of RyR2 are responsible for the generation of arrhythmias in this study. Nevertheless, these are just two of the several possible reversible redox modifications of free thiols that can occur simultaneously. The characterization of RyR2 modifications is beyond the scope of the present work.

HFD-induced cardiac hypertrophy or heart failure occurs after time periods much longer than the eight weeks used in this study [41,42,43,44]. The increase in RyR2 activity shown here is an early event induced by the HFD, that may have a role, not only in the generation of ventricular arrhythmias suggested here, but also in the long-term generation of cardiac hypertrophy induced by HFD.

## 4. Materials and Methods

### 4.1. Ethical Approval

All procedures in this work were performed in accordance to the Guide for the Care and Use of Laboratory Animals, published by the U.S. National Institutes of Health (NIH Publication, 8th Edition, 2011), and approved by the Institutional Ethics Committee of the School of Medicine, Universidad de Chile (Protocol CBA0819 FMUCH, approved on 2 June 2016).

### 4.2. Animals and Dietary Model

Male C57BL/6 mice (21 days old) were fed with a HFD (60% calories from fat, Cat N° D-12492, Research Diets, New Brunswick, NJ, USA) or with a regular diet (10% calories from fat, Champion^®^, Santiago, Chile) for 8 weeks. During this period, mice were kept at a temperature of 23 ± 2 °C and a 12:12 h light–dark cycle with free access to food and water. Some mice received apocynin (1.5 mM) in the drinking water, from the start of Week 5 to the end of Week 8. We chose Week 5 to initiate apocynin treatment because this is when differences in weight gain between control and HFD-fed mice are first noticeable. Apocynin did not cause differences in the amount of water drunk by the animals.

### 4.3. Surface Electrocardiogram (ECG)

At 8 weeks, mice were anesthetized with isoflurane (induction with 1.0–1.5% and maintenance with 0.5% in air). Electrodes were attached to the forelimbs (Lead I) and connected through an ML 136 Animal BioAmp to a PowerLab 2/26 model ML-826 data acquisition system (www.adinstruments.com). Rectal temperature was monitored and maintained at 36 ± 1 °C with the help of a heating pad. After stabilization, ECG was recorded for 5 min to quantify the occurrence of arrhythmia or tachycardia episodes.

### 4.4. Echocardiographic Determinations

Transthoracic M-mode images of the left ventricle were obtained in conscious mice with an echocardiograph equipped with an 8 MHz transducer (ATL 5000 ultrasound machine). The internal cavity sizes of the left ventricles at the end of diastole (LVIDd) and systole (LVIDs) were measured, and the fractional shortening of the left ventricle was calculated as ((LVIDd − LVIDs) /LVIDd) × 100. Mice were trained on three consecutive days previous to the echocardiographic examination as described [45].

### 4.5. Preparation of Cardiac Subcellular Fractions

After eight weeks, mice were euthanized by cervical dislocation; the hearts were harvested and cleaned of blood with Krebs solution. The ventricles were immediately frozen in liquid nitrogen to prepare whole homogenates or sarcoplasmic reticulum (SR)-enriched fractions as described in detail previously [18,46]. Heart homogenates were prepared from single hearts. SR-enriched fractions were prepared from a pool of 5 hearts.

### 4.6. Channel Recording and Analysis

RyR2 single channel activity was measured in SR-enriched fractions as previously described [18]. Briefly, the cis compartment, equivalent to cytoplasmic compartment, contained 225 mM HEPES–Tris, pH 7.4, 0.5 mM total Ca^2+^ plus sufficient *N*-(2-hydroxyethyl)-ethylenediamine-triacetic acid (HEDTA) and/or ethyleneglycol-bis(β-aminoethyl ether) *N*,*N*,*N*′,*N*′-tetraacetic acid (EGTA) to obtain the desired [Ca^2+^]; required amounts of HEDTA and/or EGTA were calculated with the WinMAXC program (www.stanford.edu/~cpatton/wmaxc.zip). The trans compartment, which is equivalent to the intrareticular compartment, contained 40 mM Ca–HEPES, 10 mM Tris–HEPES, pH 7.4. The charge carrier, therefore, was Ca^2+^. Channels were classified according to their response to cytoplasmic [Ca^2+^] [20,47]. The fraction of time that single RyR2 channels spent open (P_o_) as a function of cytoplasmic [Ca^2+^] were fitted to the following equation:

P_o_ = ((P_o max_ × [Ca^2+^]^n^)/(K_a_^n^ + [Ca^2+^]^n^)) × (K_i_/(K_i_ + [Ca^2+^])) (1)

where P_o, max_ is the theoretical P_o_ value of channel maximally activated by Ca^2+^, K_a_, and K_i_ are the Ca^2+^ concentrations for the half-maximal activation or inhibition of channel activity, respectively, and n is the Hill coefficient for channel activation by Ca^2+^.

### 4.7. Western Blot Analysis

Proteins were separated in 3.5–8% Tris-acetate SDS-PAGE gels (for RyR2) or 3.5–8% Bis-Tris SDS-PAGE gels for other proteins, transferred to polyvinylidenedifluoride (PVDF) membranes, and probed with one of the following antibodies: anti-RyR2 (ThermoScientific, Rockford, IL, USA); anti-RyR2 phosphoserine-2814 (Badrilla Ltd., Leeds, UK); anti-RyR2 phosphoserine-2808 (Badrilla Ltd., Leeds, UK); anti-NOX4 (Abcam, Cambridge, UK); anti NOX2 (BD Bioscience, Franklin Lakes, NJ, USA); anti p47phox (Sigma-Aldrich, St Louis, MO, USA); anti-phospholamban (Thermo Scientific, Rockford, IL, USA); anti-phospholamban phosphoserine-16 (Badrilla, Leeds, UK); anti-phospholamban phosphothreonine-17 (Badrilla, Leeds, UK). All Western blot determinations were performed in whole homogenates, except for the determination of p47phox, which was determined in a membrane enriched fraction.

### 4.8. Determination of RyR2 Free Thiol Content

Free SH content of RyR2 was determined by biotin labeling with EZ-link Maleimide-PEG_2_-biotin (NEM-biotin; Thermo Scientific, Rockford, IL, USA). SR vesicles were incubated with 0.2 mmol/L NEM-biotin for 120 min on ice, protected from light. Excess NEM-biotin was eliminated by incubation with 4 mmol/L glutathione (GSH) for another 30 min on ice. Proteins were then mixed with sample buffer containing urea and dithiothreitol (DTT), separated in 3.5–8% Tris-acetate SDS-PAGE gels, and transferred to PVDF membranes. Biotin content was determined by the HRP–streptavidin reaction. Results were normalized by the RyR2 content of the same sample, run in parallel in the same gel but not treated with NEM-biotin.

### 4.9. RNA Isolation and qRT-PCR

Total RNA was isolated from mouse ventricles using PureZOL^TM^ (Bio-Rad, Hercules, CA, USA) according to the manufacturer’s instructions. RNA from each sample was used for real time (RT) using the iScript cDNA synthesis kit (Bio-Rad). The cDNA was used for quantitative polymerase chain reaction (PCR) analysis in an amplification system (MX3000P, Stratagene, La Jolla, CA, USA) using Brilliant III Ultra-Fast SYBR^®^ Green QPCR Master Mix (Agilent, Santa Clara, CA, USA). Primers used for RT-PCR were as follows:

RyR2 forward 5′-CTGAGAACTGATGATGAGGTGGT-3′;
RyR2 reverse 5′-ATCCTTCTGCTGCCAAGCAC-3′;
18S forward 5′CGGACAGGATTGACAGATTG-3′;
18S reverse 5′-CAAATCGCTCCACCAACTAA-3′;
NOX4 forward 5′-TGGCCAACGAAGGGGTTAAA-3′;
NOX4 reverse 5′-ATGAGGCTGCAGTTGAGGTT-3′;
NOX2 forward 5′-CTCAGGCCAATCACTTTGCT-3′;
NOX2 reverse 5′-TTCAGGGCCACACAGGAAAA-3′.

The 2^−ΔΔ*C*t^ method was used to calculate relative transcript abundances.

### 4.10. Statistical Analysis

Data are expressed as mean ± SEM. Differences in the frequencies of arrhythmic events were assessed using the Kruskal–Wallis test followed by Dunns test. Changes in the frequency of appearance of the three different channel responses to cytoplasmic [Ca^2+^] were assessed using the chi-square test. Other data was analyzed by one-way ANOVA followed by Tukey post-test or Student’s *t*-test when comparing two groups. All statistical analysis were performed using GraphPad Prism version 5.04 for Windows, GraphPad software, La Jolla, CA, USA. Differences were considered significant at *p* < 0.05.

## 5. Conclusions

High-fat-diet-induced obesity increases the occurrence of ventricular arrhythmias and favors the increased responses of RyR2 channels to cytoplasmic Ca^2+^ concentration. Apocynin, an ROS scavenger and NADPH oxidase inhibitor, prevents the appearance of arrhythmias and the high activity response of RyR2 channels to Ca^2+^. DTT, a thiol reducing agent, transforms high-activity RyR2 channels into moderate activity channels. Both the effect of apocynin and DTT suggest that the increased response RyR2 channels to Ca^2+^ is due to the redox modifications of the protein. Obesity increases the expression of NOX4; this ROS-generating enzyme could be responsible for the redox modification of RyR2 in obese mice.

## Figures and Tables

**Table 1:** Heart rate and fractional shortening in conscious mice.

*n*	Control 10	HFD 10	Control with Apocynin 8	HFD with Apocynin 8
Heart rate (BPM)	668 ± 113	778 ± 142 *	637 ± 83	745 ± 70 *
LVID diastole, mm	1.58 ± 0.27	1.66 ± 0.58	1.74 ± 0.17	1.74 ± 0.34
LVID systole, mm	0.78 ± 0.17	0.77 ± 0.32	0.90 ± 0.18	0.96 ± 0.30
FS %	49 ± 12	50 ± 16	47 ± 14	51 ± 11

LVID: Left ventricular internal diameter; FS: fractional shortening; HFD: high fat diet. Values are given as mean ± SD. * *p* < 0.05 compared to control.

**Table 2:** Fitting parameters of the three responses to cytoplasmic [Ca^2+^] of single RyR2 channels.

Channel Activity	K_a_ (µM)	n_Hill_	K_i_ (µM)
Low	47 ± 16	1.5 ^#^	7.0 ± 2.4
Moderate	9.3 ± 1.7 *	1 ^#^	177 ± 28 ^$^
High	1.1 ± 0.1 *^,$^	3 ^#^	5000 ^#^

^1^ Values were obtained from the best nonlinear fit to Equation (1) of the values obtained with single channels from control hearts that displayed low, moderate, or high activity responses. * *p* < 0.05 vs. moderate or low; ^$^ *p* < 0.001 vs. low. ^#^ Parameter was fixed to the indicated value for data fitting.

## Supplementary Materials

Supplementary materials can be found at http://www.mdpi.com/1422-0067/19/2/533/s1.
