# Supplementary material for: High-Fat-Diet-Induced Obesity Produces Spontaneous Ventricular Arrhythmias and Increases the Activity of Ryanodine Receptors in Mice
Source: Int J Mol Sci. 2018 Feb 10;19(2):533. doi: 10.3390/ijms19020533 (PMC5855755; doi:10.3390/ijms19020533)
Supplement: Supplementary file 1 [file ijms-19-00533-s001.pdf]

Table S1. Effect of HFD on Body Weight, Glycemia, plasma Triglycerides and total plasma Cholesterol.

	**Control**	**HFD**	**Control + Apo**	**HFD + Apo**
Body Weight, g	23.5 ± 1.0 (11)	31.6 ± 1.9 * (12)	24.2 ± 1.4 (7)	29.5 ± 2.7 * (9)
Glycemia g/dL	133.8 ± 20.2 (11)	215.2 ± 49.5 * (12)	152.8 ± 10.8 (7)	212.8 ± 48.2 (9)
Triglycerides mg/dL	70.0 ± 15.5 (6)	78.3 ± 15.3 (6)	61.3 ± 5.6 (6)	68.5 ± 11.5 (6)
Total Cholesterol mg/dL	75.3 ± 14.6 (6)	117.6 ± 6.8 * (6)	82.4 ± 11.1 (6)	95.9 ± 6.2 * # (6)

Values are the Mean ± SD of the indicated number of mice and were obtained after 6 h of fasting. * $p < 0.05$, compared to control; # $p < 0.05$, compared to HFD. Determinations were made with enzymatic colorimetric tests from Human, Wiesbaden-Germany.
